# Broad-spectrum capture of clinical pathogens using engineered Fc-mannose-binding lectin enhanced by antibiotic treatment

**DOI:** 10.12688/f1000research.17447.1

**Published:** 2019-01-25

**Authors:** Benjamin T. Seiler, Mark Cartwright, Alexandre L. M. Dinis, Shannon Duffy, Patrick Lombardo, David Cartwright, Elana H. Super, Jacqueline Lanzaro, Kristen Dugas, Michael Super, Donald E. Ingber

**Affiliations:** 1 Wyss Institute for Biologically Inspired Engineering, Harvard University, Boston, Massachusetts, 02115, USA; 2 Vascular Biology Program, Boston Children's Hospital and Harvard Medical School, Boston, Massachusetts, 02115, USA; 3 Harvard John A. Paulson School of Engineering and Applied Sciences, Harvard University, Cambridge, Massachusetts, 02138, USA

**Keywords:** Mannose-binding lectin, Lipopolysaccharide, Lipoteichoic acid, Biomarker, Bacteria, Antibiotics, Diagnostic

## Abstract

**Background:** Fc-mannose-binding lectin (FcMBL), an engineered version of the blood opsonin MBL that contains the carbohydrate recognition domain (CRD) and flexible neck regions of MBL fused to the Fc portion of human IgG1, has been shown to bind various microbes and pathogen-associated molecular patterns (PAMPs). FcMBL has also been used to create an enzyme-linked lectin sorbent assay (ELLecSA) for use as a rapid (<1 h) diagnostic of bloodstream infections.

**Methods:** Here we extended this work by using the ELLecSA to test FcMBL’s ability to bind to more than 190 different isolates from over 95 different pathogen species.

**Results: **FcMBL bound to 85% of the isolates and 97 of the 112 (87%) different pathogen species tested, including bacteria, fungi, viral antigens and parasites. FcMBL also bound to PAMPs including, lipopolysaccharide endotoxin (LPS) and lipoteichoic acid (LTA) from Gram-negative and Gram-positive bacteria, as well as lipoarabinomannan (LAM) and phosphatidylinositol mannoside 6 (PIM _6_) from *Mycobacterium tuberculosis*.

**Conclusions:** The efficiency of pathogen detection and variation between binding of different strains of the same species could be improved by treating the bacteria with antibiotics, or mechanical disruption using a bead mill, prior to FcMBL capture to reveal previously concealed binding sites within the bacterial cell wall. As FcMBL can bind to pathogens and PAMPs in urine as well as blood, its broad-binding capability could be leveraged to develop a variety of clinically relevant technologies, including infectious disease diagnostics, therapeutics, and vaccines.

## Introduction

Mannose-binding lectin (MBL) is a key host-defense protein associated with the lectin pathway of the innate immune system ^1^, and deficiency of MBL can lead to increased susceptibility to a wide-spectrum of infectious diseases ^2– 4^. MBL functions as a calcium-dependent, pattern-recognition opsonin that binds a range of carbohydrate molecules associated with the surfaces or cell walls of many different types of pathogens ^5^. Collectively these microbial surface carbohydrate molecules, including for example, lipopolysaccharide endotoxin (LPS) and lipoteichoic acid (LTA), are referred to as pathogen-associated molecular patterns (PAMPs) ^6, 7^. MBL has the intrinsic ability to distinguish foreign PAMPs from self, subsequently activating the complement system and providing protection via antibody-dependent and independent mechanisms ^8, 9^.

 Due to the evolutionarily conserved recognition carbohydrate moieties of PAMPs, MBL is a broad-spectrum opsonin that can bind over 90 different species of pathogens, including Gram-negative and Gram-positive bacteria, fungi, viruses, and parasites ^10– 14^. MBL binding to these various pathogens has been demonstrated by means of flow cytometry ^14, 15^, radio-immunoassay ^13, 16^, enzyme-linked immunosorbent assay (ELISA) ^13, 17^, immunofluorescence and scanning electron microscopy (SEM) ^18^, and *Saccharomyces cerevisiae*-induced MBL activation and bystander lysis of chicken erythrocytes ^19^. However, many discrepancies in MBL binding have been described, depending on the method used. For example, use of flow cytometry revealed little to no MBL binding to *Pseudomonas aeruginosa*, while others have reported good binding of MBL to *Pseudomonas aeruginosa* using a hemolytic assay ^15, 19^ *.*

 We set out to address these conflicting results by leveraging the recent development of an engineered version of MBL that contains the carbohydrate recognition domain (CRD) and flexible neck regions of MBL fused to the Fc portion of human IgG1, which is known as FcMBL ^20^. The engineered FcMBL lacks the regions of the native molecule that interact with MBL-associated serine proteases (MASPs) that activate complement and promote blood coagulation, and thus, it can be used to capture PAMPs from complex biological fluids, such as blood and urine, without activating effector functions of complement, coagulation, and phagocytosis. We have previously used FcMBL in extracorporeal therapies, such as hemofiltration, and in diagnostics to capture and detect *Staphylococcus aureus* from osteoarticular and synovial fluids of infected patients ^20– 22^. In the present study, we used a previously described sandwich enzyme-linked lectin sorbent assay (ELLecSA) in which both live and fragmented pathogens (PAMPs) are captured using FcMBL conjugated to magnetic beads and then detected with horseradish peroxidase (HRP)-labeled MBL ^23^. This ELLecSA has demonstrated FcMBL binding to 85% (47 of 55) of pathogen species previously tested, and enabled rapid diagnosis of bloodstream infections by capturing and detecting PAMPs in whole blood from human patients ^23^. Here we extend this testing to include 69 isolates from 57 more pathogen species using the ELLecSA. In total, we measure direct binding of FcMBL to over 190 pathogen isolates from over 95 different pathogen species, including bacteria, fungi, viral antigens, parasites, and bacterial cell wall molecules. As a result of this more extensive analysis, we demonstrate that FcMBL detection increases to 85% of the isolates and 87% (97 out of 112) of the pathogen species tested. Furthermore, we show that antibiotic treatment or mechanical disruption of the bacterial pathogens exposes previously concealed FcMBL binding sites on cell walls, thereby increasing the efficiency of pathogen detection and reducing variation between binding of different strains of the same species. We also show that FcMBL can detect PAMPs in urine as well as blood, making this potential diagnostic technology highly synergistic with standard of care antibiotic therapy.

## Results

### FcMBL binding to bacteria

**Figure 1.**
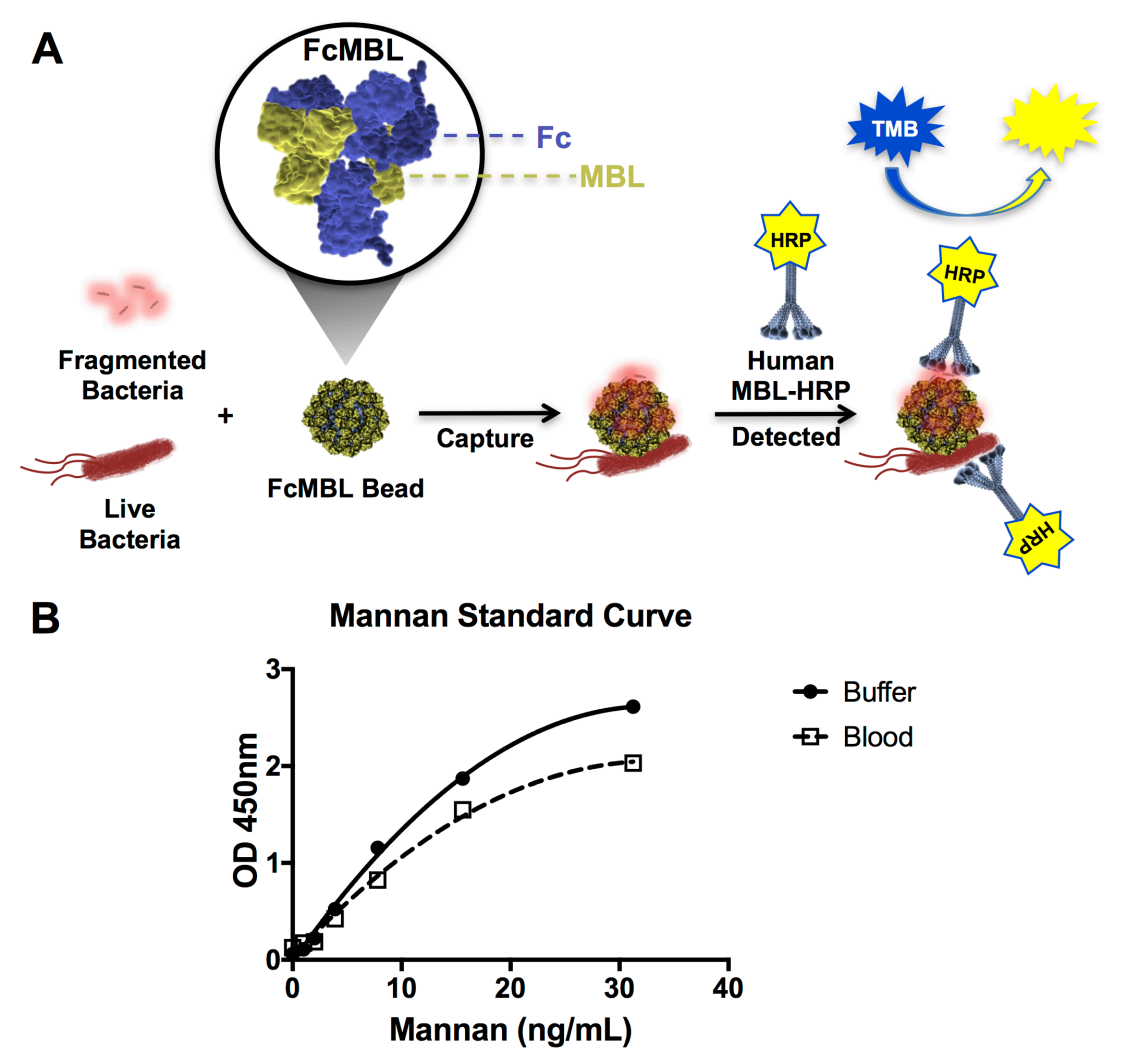
FcMBL ELLecSA. ( **A**) Diagrammatic representation of the FcMBL ELLecSA methodology. Live and fragmented bacteria are captured by FcMBL, which has been coated on superparamagnetic beads via an N-terminal aminooxy-biotin on the Fc to allow oriented attachment to streptavidin (FcMBL Bead). FcMBL beads with captured bacteria are then magnetically separated and detected using recombinant human MBL linked to horseradish peroxidase (Human MBL-HRP). Tetramethylbenzidine (TMB) substrate is added to quantify captured bacteria and results are read at OD 450nm. ( **B**) Mannan standard curve showing FcMBL binding to mannan in buffer and spiked into whole human blood (<24 h from draw), measured at optical density 450 nm. The mannan standard curve in buffer serves as an internal assay control, and is used to determine FcMBL binding to pathogen material in units of PAMPs/ml (where 1 ng/ml of mannan bound by FcMBL is equivalent to 1 PAMP unit. PAMP units are then multiplied by the dilution factor of the sample volume to give PAMPs/ml).

We first set out to determine the range of pathogens that FcMBL can capture by screening multiple species of bacteria, fungi, viral antigens, and parasites using the ELLecSA detection technology. In the FcMBL ELLecSA, pathogen materials in experimental samples are captured with FcMBL immobilized on superparamagnetic beads (1 µm diameter), magnetically separated, washed, detected with human MBL linked to horseradish peroxidase (HRP), magnetically separated again, washed, and then tetramethylbenzidine (TMB) substrate is added to measure the amount of pathogen material bound ( Figure 1A). Results were quantified by interpolating against an internal standard curve generated using yeast mannan in buffer [50 mM Tris-HCl, 150 mM NaCl, 0.05% Tween-20, 5 mM CaCl _2_, pH 7.4 (TBST 5 mM CaCl _2_)] ( Figure 1B). In addition, we show that while the curve sensitivity is reduced when mannan is spiked into whole human blood, the limit of detection remained similar (1 ng/ml) as it does in buffer ( Figure 1B).

Initially our focus was on screening bacteria and as such we compiled a comprehensive list of clinically relevant bacterial pathogens ( Table 1) ^23^. When we screened 82 different species of bacteria to compare FcMBL binding to live versus fragmented cells, we found that FcMBL detected 59 out of 82 live microbes (72%) and that more could be detected (70 out of 82; 85%) after they were fragmented by treating with antibiotics, or mechanically disrupted using zirconia/silica beads in a mixer mill ( Table 1) ^23^. The antibiotics we used in this study were clinical grade cefepime, ceftriaxone, meropenem, amikacin, gentamicin, and vancomycin, to provide enough coverage to target this diverse range of bacteria. We dosed each bacterial class with a single appropriate antibiotic dose (≤1 mg/ml) to obtain acute fragmentation within 4 hours.

**Table 1.** FcMBL binding profile of bacteria, fungi, viral antigens, parasites, and bacterial antigens. Multiple species of bacteria, including multiple isolates (number of isolates), were screened by FcMBL ELLecSA to determine FcMBL binding. Total number detected of both live and fragmented bacterial isolates is shown. Fungi were screened and total number detected for live isolates shown. Purified or inactivated viral antigens, parasites, and bacterial antigens were tested directly in TBST 5 mM CaCl _2_ buffer, and number detected shown. Test samples were performed in duplicate. NT, not tested.

Bacteria					Fungi			
Genus	Species	# of Isolates	Live Detected	Fragmented Detected	Genus	Species	# of Isolates	Live Detected
*Acinetobacter*	*calcoaceticus*	1	0	1	*Aureobasidium*	*pullulans*	1	1
*Acinetobacter*	*lwoffii*	1	0	1	*Candida*	*auris*	1	1
*Achromobacter*	*xylosoxidans*	1	1	1	*Penicillium*	*crustosum*	1	1
*Actinobacillus*	*pleuropneumoniae*	1	1	1				
*Aerococcus*	*viridans*	1	1	1	Viral Antigens			
*Aeromonas*	*hydrophila*	1	1	1				
*Aeromonas*	*veronii*	1	1	1	Species	Antigen	# of Isolates	Detected
*Alcaligenes*	*faecalis*	1	0	1				
*Bacillus*	*anthracis*	1	1	1	*Chikungunya*	*E1*	1	0
*Bacillus*	*cereus*	1	1	1	*Cytomegalovirus*	*particles*	1	1
*Bacillus*	*thurigiensis*	1	0	1	*Dengue*	*Serotype 1 VLP*	1	1
*Bartonella*	*henselae*	1	1	1	*Ebola*	*GP1*	1	1
*Cardiobacterium*	*hominis*	1	1	1	*HIV*	*gp120*	1	1
*Corynebacterium*	*amycolatum*	1	1	1	*Influenza H1N1*	*hemagglutinin*	1	1
*Corynebacterium*	*minutissmum*	1	1	1	*Influenza H1N1*	*neuraminidase*	1	1
*Corynebacterium*	*pseudodiphtheriticum*	1	1	1	*Respiratory Syncytial*	*glycoprotein g*	1	1
*Corynebacterium*	*striatum*	1	1	1	*Tick-borne Encephalitis*	*NS1*	1	0
*Citrobacter*	*koseri*	1	0	1	*Zika*	*lysate*	1	1
*Eikenella*	*corrodens*	1	1	1				
*Enterococcus*	*avium*	1	1	1	Parasites			
*Enterococcus*	*casseliflavus*	1	1	1				
*Escherichia*	*coli*	2	0	2	Genus	Species	# of Isolates	Detected
*Kingella*	*kingae*	1	0	0				
*Lactococcus*	*lactis*	1	1	1	*Plasmodium*	*falciparum*	1	0
*Laribacter*	*hongkongensis*	1	1	1	*Trichomonas*	*vaginalis*	1	1
*Moraxella*	*catarrhalis*	1	1	1				
*Mycobacterium*	*bovis*	1	NT	1	Bacterial Antigens			
*Providencia*	*stuartii*	1	1	1				
*Pseudomonas*	*aeruginosa*	5	5	5	Antigen		# of Isolates	Detected
*Pseudomonas*	*putida*	1	1	1				
*Pseudomonas*	*stutzeri*	1	0	1	Lipoarabinomannan		1	1
*Rothia*	*mucilaginosa*	1	0	1	Lipopolysaccharide		5	5
*Staphylococcus*	*aureus* (MRSA)	1	1	1	Lipomannan		1	1
*Staphylococcus*	*caprae*	1	0	0	Lipoteichoic acid		3	3
*Staphylococcus*	*cohnii*	1	1	1	Phosphatidylinositol mannoside 1,2		1	0
*Staphylococcus*	*haemolyticus*	1	0	0	Phosphatidylinositol mannoside 6		1	1
*Staphylococcus*	*intermedius*	1	1	1				
*Staphylococcus*	*warneri*	1	0	0				
*Streptococcus*	*anginosus*	1	0	1				
*Streptococcus*	*bovis*	1	1	1				
*Streptococcus*	*Group B*	1	1	1				
*Streptococcus*	*Group C*	1	1	1				
*Streptococcus*	*dysgalactiae*	1	1	1				
*Streptococcus*	*pneumoniae*	4	4	4				
*Streptococcus*	*sanguinis*	1	1	1				
*Streptococcus*	*suis*	1	0	1				

**Figure 2.**
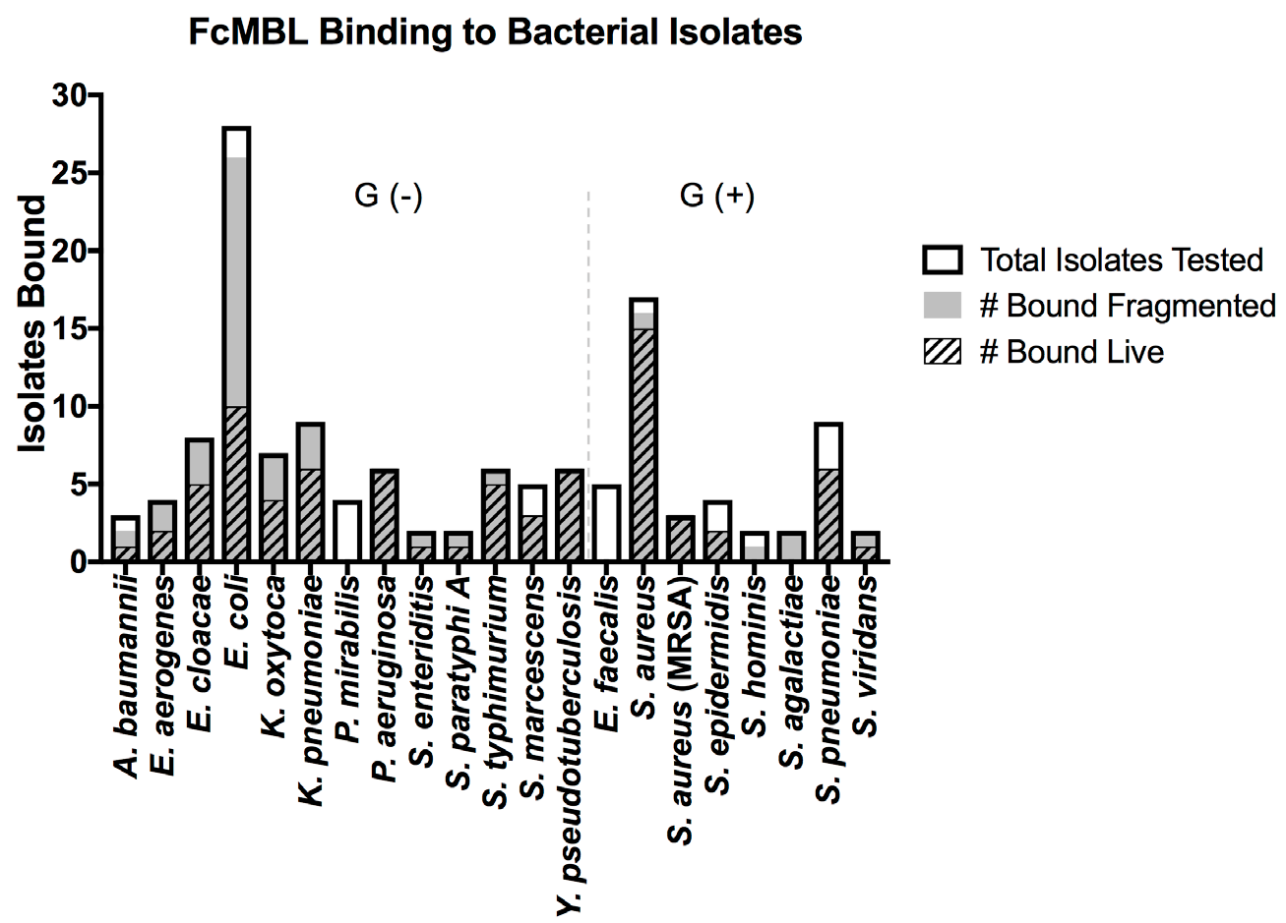
FcMBL binding to bacterial isolates is equal to or enhanced upon fragmentation. The graph is divided between Gram-negative bacterial isolates [Gram (-)] and Gram-positive bacterial isolates [Gram (+)]. Data are presented as the number of bacterial isolates bound live and fragmented within the total isolates tested for each species: *A. baumannii* ( *n* = 3), *E. aerogenes* ( *n* = 4), *E. cloacae* ( *n* = 8), *E. coli* ( *n* = 28), *K. oxytoca* ( *n* = 7), *K. pneumoniae* ( *n* = 9), *P. mirabilis* ( *n* = 4), *P. aeruginosa* ( *n* = 6), *S. enteriditis* ( *n* = 2), *S. paratyphi A* ( *n* = 2), *S. typhimurium* ( *n* = 6), *S. marcescens* ( *n* = 5), *Y. pseudotuberculosis* ( *n* = 6), *E. faecalis* ( *n* = 5), *S. aureus* ( *n* = 17), *S. aureus* (MRSA) ( *n* = 3), *S. epidermidis* ( *n* = 4), *S. hominis* ( *n* = 2), *S. agalactiae* ( *n* = 2), *S. pneumoniae* ( *n* = 9), *S. viridans* ( *n* = 2).

To determine if inducing bacterial fragmentation via antibiotic treatment or bead milling would reduce variation in FcMBL binding between different strains of the same species, we screened 134 isolates from 21 of the 88 Gram-positive and Gram-negative bacterial species, including strains tested by Cartwright *et al*. ( Figure 2) ^23^. As before, FcMBL bound a greater proportion of the pathogens when fragmented (113/134 = 84%) than when live and intact (77/134 = 57%). For some bacterial species such as *Enterobacter cloacae*, *Escherichia coli*, *Klebsiella oxytoca*, and *Klebsiella pneumoniae* we found that mechanical disruption or antibiotic-induced fragmentation greatly increased FcMBL binding, whereas other bacteria like *Pseudomonas aeruginosa*, *Yersinia pseudotuberculosis*, and MRSA bound equally well when live and intact ( Figure 2). With the exception of *Proteus mirabilis* and *Enterococcus faecalis*, which FcMBL did not bind at all, the capture of fragmented bacteria was equal to or greater than that of live bacteria ( Figure 2).

**Figure 3.**
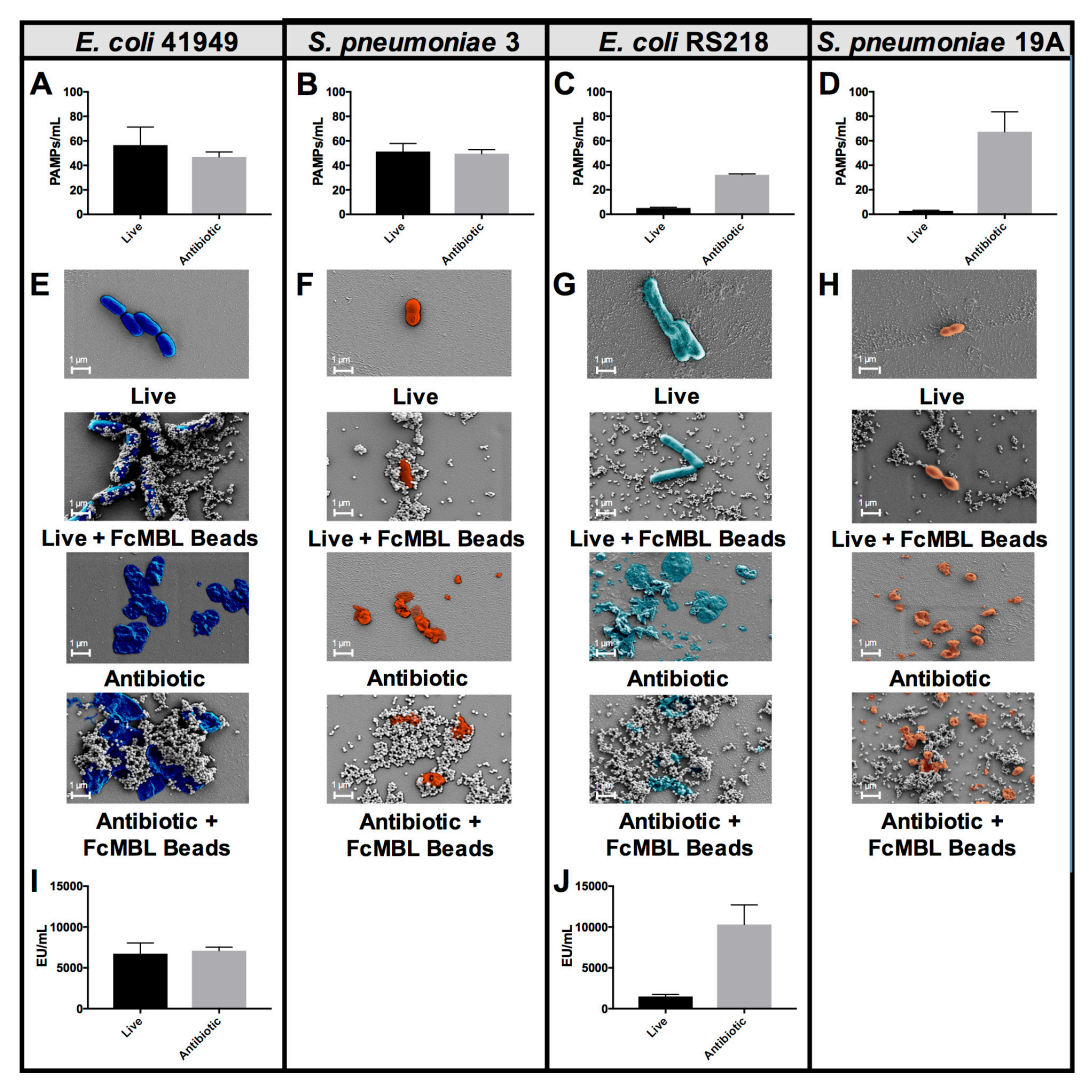
FcMBL bacterial binding efficiency can be enhanced with antibiotic treatment. ( **A**– **D**) PAMPs/ml detection by FcMBL ELLecSA of both live (10 ^7^ CFU/ml) and fragmented bacteria using antibiotics (cefepime 1 mg/ml or ceftriaxone 1 mg/ml). ( **A**) *E. coli* 41949, ( **B**) *S. pneumoniae* 3, ( **C**) *E. coli* RS218, and ( **D**) *S. pneumoniae* 19A. ( **E**– **H**) Scanning electron microscopy images showing FcMBL bead (128 nm) capture of both live and fragmented bacteria using antibiotics (cefepime 1 mg/ml or ceftriaxone 1 mg/ml). ( **E**) *E. coli* 41949, ( **F**) *S. pneumoniae* 3, ( **G**) *E. coli* RS218, and ( **H**) *S. pneumoniae* 19A. ( **I**, **J**) LPS endotoxin measurement (LAL assay) using 10 ^7^ CFU/ml of both live and fragmented bacteria using antibiotics (cefepime 1 mg/ml). ( **I**) *E. coli* 41949 and ( **J**) *E. coli* RS218.

These findings are consistent with past studies that showed the efficiency of MBL binding to live bacteria differs between isolates from the same bacterial genus and species, possibly due to differences in encapsulation ^15, 16^. To illustrate that the heterogeneity of MBL binding to live isolates of the same species can be reduced by using antibiotics to disrupt previously cryptic binding sites, we used two different isolates of *Escherichia coli*, and two different isolates of *Streptococcus pneumoniae*. *E*. *coli* 41949 and *S*. *pneumoniae* 3 exhibited equivalent FcMBL binding whether they were live or fragmented with antibiotics (1 mg/ml cefepime or ceftriaxone, respectively, for 4 hours), whereas fragmented forms of *E. coli* RS218 and *S. pneumoniae* 19A isolates bound much more effectively to FcMBL than living forms ( Figure 3A–D). This difference was further supported visually using scanning electron microscopy (SEM) in which magnetic FcMBL beads could be seen to bind both live and fragmented versions of *E. coli* 41949 and *S. pneumoniae* 3, but with *E. coli* RS218 and *S. pneumoniae* 19A, the FcMBL beads only bound to fragmented material ( Figure 3E–H). FcMBL binding increases upon fragmentation, which is correlated with LPS release measured using a limulus amebocyte lysate (LAL) assay: equal amounts of LPS were detected for *E. coli* 41949 whether live or fragmented, whereas LPS levels were higher in antibiotic treated *E. coli* RS218 ( Figure 3I, J). These results suggest that antibiotic treatment results in exposure of previously cryptic PAMPs in the cell wall, including toxins such as LPS and LTA, which leads to greatly increased binding of FcMBL.

**Figure 4.**
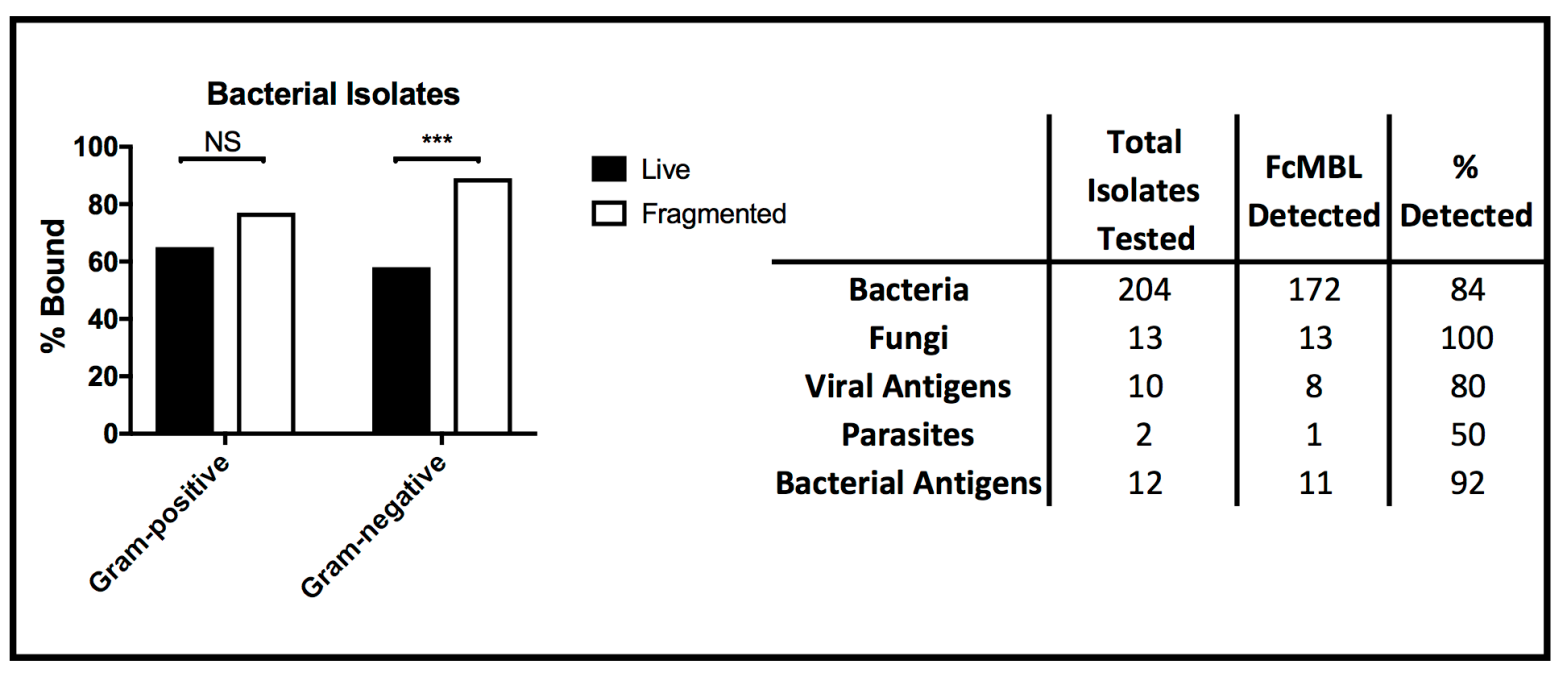
Summary of FcMBL pathogen capture. (Left) Percent of live and fragmented Gram-positive ( *n* = 78) and Gram-negative ( *n* = 117) bacterial isolates bound by FcMBL ELLecSA. (Right) Chart showing total number of isolates tested by FcMBL ELLecSA for bacteria, fungi, viral antigens, parasites, and bacterial antigens, total number FcMBL detected, and total percent detected overall. ***p-value < 0.0001; Pearson’s chi-squared test. NS, not significant.

In these studies, we found that 12 bacterial species, including multiple species of *Enterococcus and Proteus*, failed to bind to FcMBL even when treated for 4 hours with combinations of antibiotics (500 µg/ml vancomycin and 500 µg/ml amikacin for Gram-positive isolates or 500 µg/ml cefepime and 500 µg/ml amikacin for Gram-negative isolates) ( Table 1) ^23^. Importantly however, FcMBL was able to detect 84% (172 out of 204) of the bacterial isolates ( Figure 4), which includes 9 of the 10 pathogens responsible for most healthcare-associated infections in acute care hospitals in the U.S., with *Enterococcus* species being the one exception ^24^.

### FcMBL binding to bacterial cell wall components

We further explored FcMBL’s ability to bind bacterial cell wall components because when bead mill or antibiotics were used to disrupt the membranes of Gram-negative isolates ( *n* = 117), there was a significant boost in FcMBL detection efficiency with fragmented cells (89%) versus live intact cells (58%) ( Figure 4), which is likely due to exposure of LPS that is present in high concentrations in their cell wall ^25^. Similarly, FcMBL also detected a greater percentage (77%) of fragmented Gram-positive isolates ( *n* = 78), versus live intact cells (65%) ( Figure 4). Thus, to better understand some of the major targets that FcMBL binds when bacteria are fragmented, we extended our analysis using purified samples of LPS and LTA ^25, 26^.

**Figure 5.**
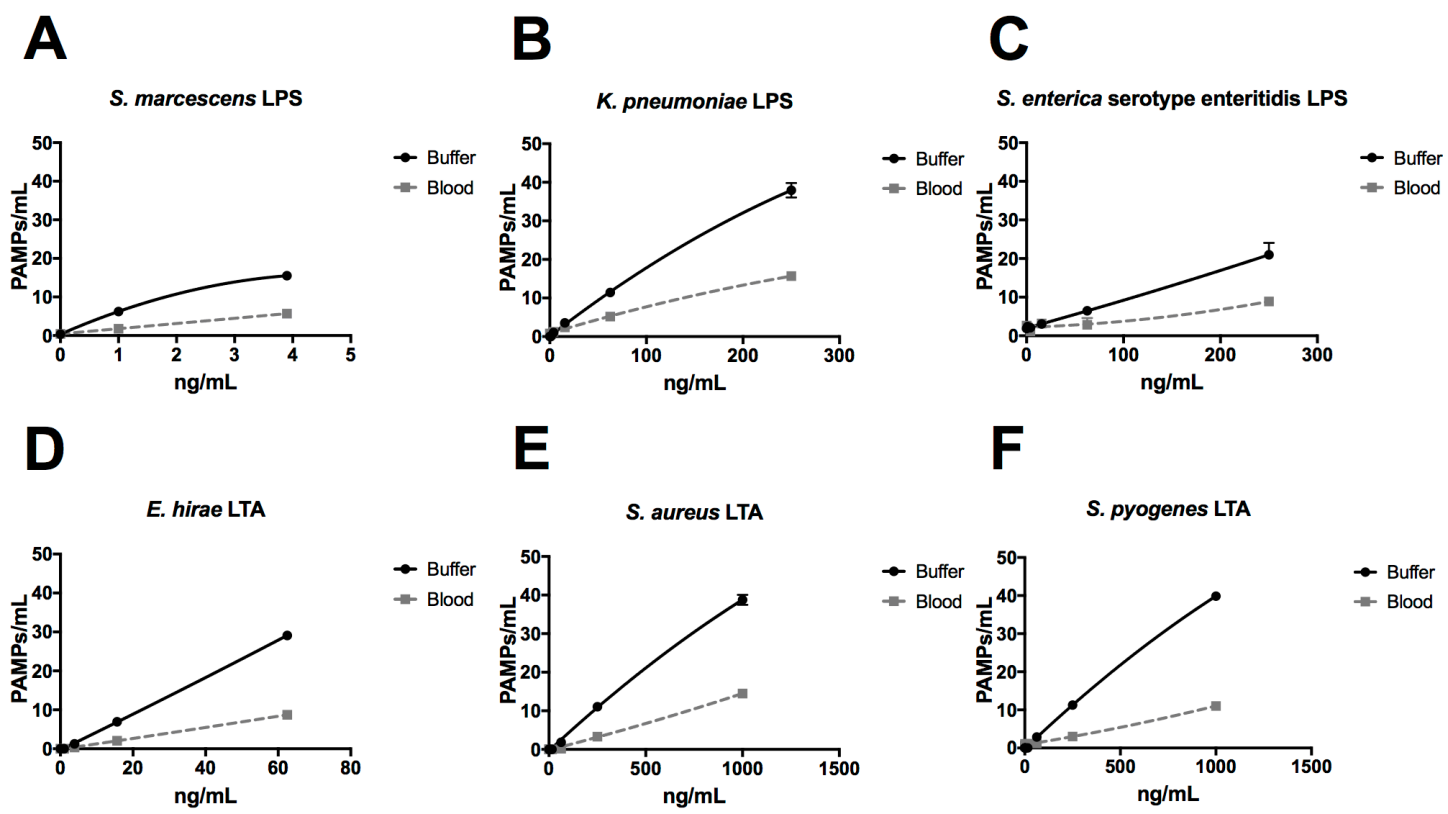
FcMBL ELLecSA screening of purified lipopolysaccharide (LPS) and lipoteichoic acid (LTA). LPS from ( **A**) *S. marcescens*, ( **B**) *K. pneumoniae*, and ( **C**) *S. enterica* serovar enteritidis, and LTA from ( **D**) *E. hirae*, ( **E**) *S. aureus*, and ( **F**) *S. pyogenes* were spiked into either TBST 5 mM CaCl _2_ buffer or whole human blood at indicated concentrations.

Using the ELLecSA, we screened LPS purified from Gram-negative bacteria ( *Serratia marcescens*, *Klebsiella pneumoniae*, and *Salmonella enterica* serovar enteritidis), as well as LTA from Gram-positive bacteria ( *Enterococcus hirae*, *Staphylococcus aureus*, and *Streptococcus pyogenes*) in TBST 5 mM CaCl _2_, as well as in more clinically relevant human whole blood samples. We found that FcMBL was able to detect LPS from all 3 Gram-negative species, with *S. marcescens* being the best (1 ng/ml limit of detection in buffer and 3.9 ng/ml in blood) ( Figure 5A–C). FcMBL also bound to *E. hirae* LTA very well (15.6 ng/ml limit of detect in buffer and 62.5 ng/ml in blood) ( Figure 5D), which is consistent with past findings ^27^. Also consistent with past findings, we found that FcMBL binds *S. aureus* LTA through the carbohydrate recognition domain ( Figure 5E and Supplementary Figure 1) ^28, 29^. Notably, however, FcMBL also bound *S. pyogenes* LTA ( Figure 5F), which is in contrast to the past finding that MBL binds well to *E. hirae* LTA but poorly to LTA from *S. pyogenes* due to lack of glycosyl substituents ^27, 30^.

**Figure 6.**
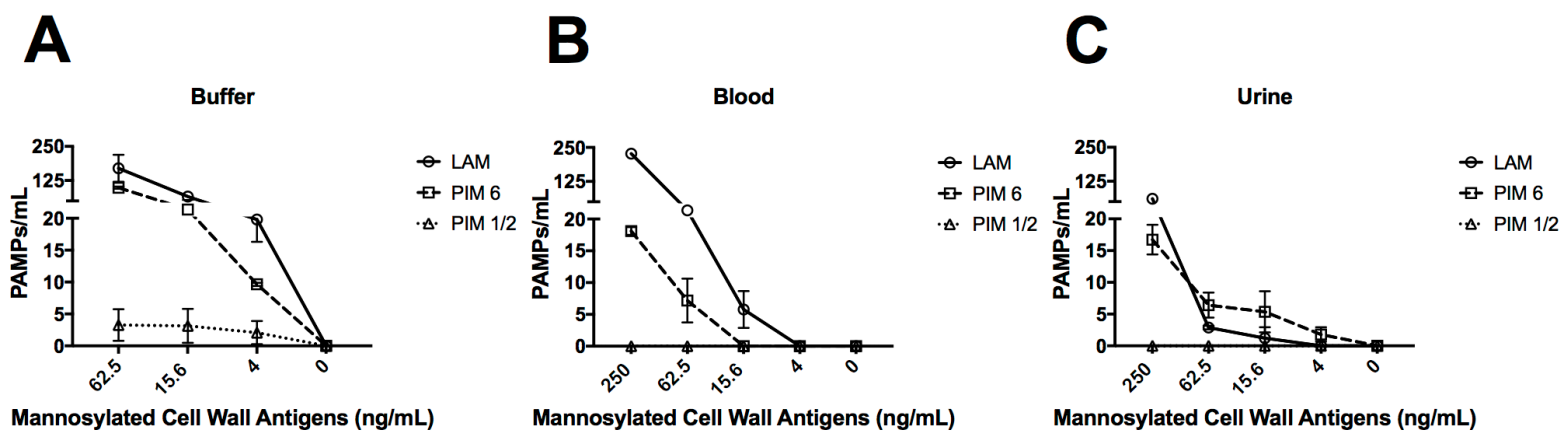
FcMBL ELLecSA screening of *Mycobacterium tuberculosis* strain H37Rv glycolipids PIM _1,2_, PIM _6_, and LAM. Glycolipids spiked into ( **A**) buffer (TBST 5mM CaCl _2_), ( **B**) whole human blood, and ( **C**) urine. FcMBL detected LAM and PIM _6_ at 4 ng/ml in buffer, but not PIM _1,2_. Sensitivity of LAM and PIM _6_ is reduced in whole human blood and urine.

We next tested FcMBL’s ability to bind lipoarabinomannan (LAM) and its biosynthetic precursors, phosphatidylinositol mannoside 1 & 2 and 6 (PIM _1,2_ and PIM _6_) from *Mycobacterium tuberculosis* (TB) strain H37Rv ^31, 32^. LAM released from metabolically replicating or degrading TB bacteria has been detected in both blood and urine ^33, 34^. Thus, we assessed the ability of FcMBL to capture and detect LAM, as well as PIM _1,2 _and PIM _6_, spiked into both of these complex biological fluids as well as buffer. Our initial screen in buffer confirmed that FcMBL can detect LAM and PIM _6 _at levels down to 4 ng/ml, but it did not detect PIM _1,2 _( Figure 6A–C). FcMBL also bound to LAM in both blood and urine but its binding sensitivity was reduced as it could only detect 15.6 ng/ml and 250 ng/ml, respectively. FcMBL binding to PIM _6_ exhibited a similar sensitivity in buffer, but it could only detect 62.5 ng/ml and 15.6 ng/ml in blood and urine, respectively.

### FcMBL binding to fungi, parasites, viral antigens, and bacterial cell wall antigens

**Figure 7.**
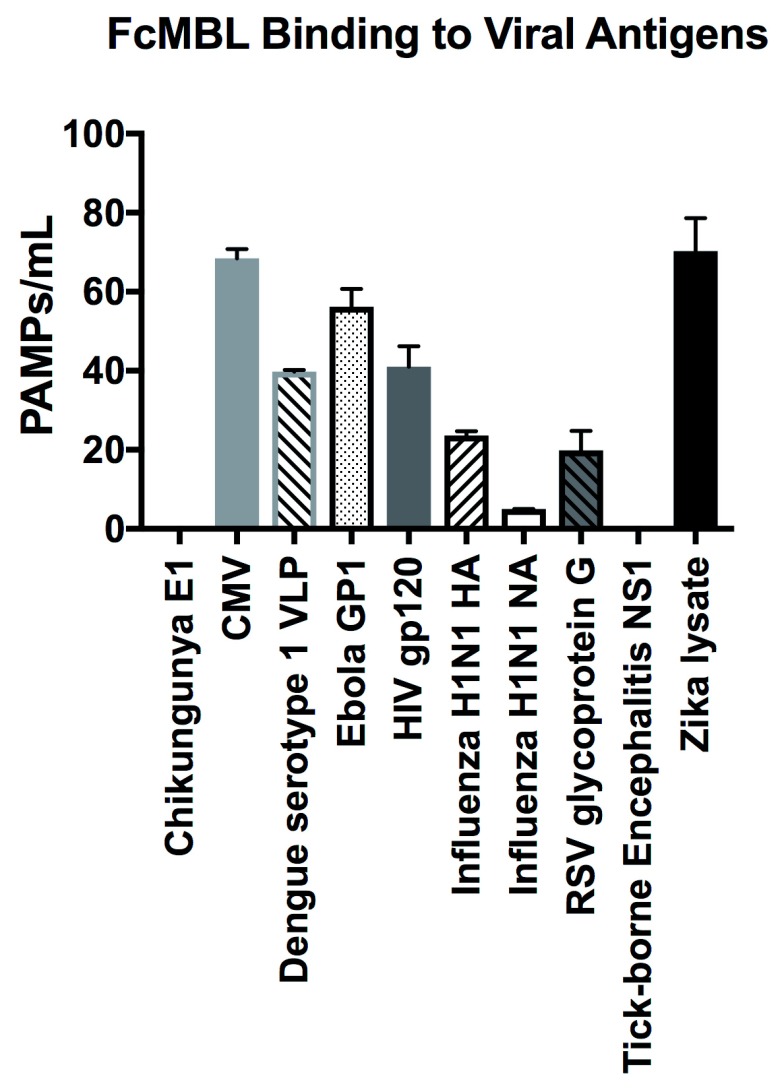
FcMBL binding to viral antigens. FcMBL ELLecSA screening of Chikungunya E1 (3.0 µg/ml), Cytomegalovirus (CMV) (10 ^7^ PFU), Dengue serotype 1 VLP (0.36 µg/ml), Human immunodeficiency virus (HIV) gp120 (0.1 µg/ml), Ebola GP1 (0.2 µg/ml), Influenza H1N1 HA (0.1 µg/ml), Influenza H1N1 NA (1 µg/ml), Respiratory syncytial virus (RSV) glycoprotein g (10.0 µg/ml), Tick-borne Encephalitis NS1 (5 µg/ml), and Zika lysate (0.24 µg/ml).

In addition to screening multiple bacteria, we also tested FcMBL’s ability to bind to 12 different species of fungi, 10 viral antigens, 2 species of parasites, and 6 purified bacterial cell wall antigens ( Table 1) ^23^. In contrast to studies with bacteria, FcMBL was found to bind 100% of live fungal cells from all 12 species and 13 isolates tested ( Figure 4). Of the two parasites tested in this preliminary analysis, only *Trichomonas vaginalis* was bound by FcMBL, whereas 80% of the viral antigens screened and 92% of the purified bacterial cell wall antigens were detected ( Figure 4 and Figure 7). The handful of pathogen material FcMBL did not detect included the E1 protein from Chikungunya virus, the NS1 protein from Tick-borne encephalitis virus, *Plasmodium falciparum*, and PIM _1,2 _from TB. In total, the overall FcMBL binding profile respectively detected 85% (194 out of 229) of isolates, and 87% (97 out of 112) of the different pathogen species tested.

Raw data for the present study are available on OSF ^29^.

## Discussion

MBL has been reported to bind to over 90 different pathogen species as well as PAMPs released from these microbes based on studies in which binding was assessed by means of flow cytometry, ELISA, radio-immunoassay, immunofluorescence and SEM, or hemolytic assays ^12– 19^; however, different results have been obtained with different methods. Here we explored the broad-spectrum binding capabilities of an engineered form of MBL, known as FcMBL, using a previously described magnetic ELLecSA detection assay to quantify binding of MBL to over 190 isolates from over 95 different pathogen species, which include bacteria, fungi, viral antigens, parasites, and bacterial cell wall antigens ^23^. FcMBL was previously shown to bind PAMPs released from 47 of 55 (85%) pathogen species tested, including 38 species of bacteria and 9 species of fungi ^23^. The FcMBL ELLecSA also was able to detect infectious PAMPs in whole blood of sepsis patients, regardless of antibiotic therapy (blood culture positive or negative) with a detection sensitivity and specificity of 85% and 89%, respectively ^23^. In the present study, we utilized the ELLecSA to extend this testing to include 69 isolates from 57 more pathogen species. In total, our results confirm that FcMBL binds to 85% of the isolates and 97 of the 112 species tested, which corresponds to an increased detection sensitivity of 87%.

 MBL binding to different clinical bacterial isolates of the same species has previously produced conflicting results ^15, 16^. These same studies also described that most Gram-negative isolates (encapsulated strains) bound little or no MBL. We have reported similar results as we previously found that FcMBL only bound 38% of live clinical *E. coli* isolates tested; however, upon fragmentation and release of PAMPs, FcMBL detection of these same isolates increased to 92% ^23^. Broader examination of Gram-negative bacteria in the present study revealed a similar pattern: FcMBL only detected 68/117 (58%) of live isolates, but when these same microbes were treated with antibiotics or bead mill, the detection sensitivity significantly increased to 89% (104/117 isolates). Apparently, by treating bacteria with antibiotics or bead mill we were able to disrupt the encapsulated cell wall, exposing and presenting previously hidden PAMPs, thereby increasing binding and reducing variability between isolates within the same bacterial species. However, even with cell wall disruption, FcMBL did not bind 12 bacterial species, including multiple isolates of *E. faecalis* and *P. mirabilis*. These microbes likely lack the complex polysaccharide antigens which FcMBL and MBL bind. Alternatively, the binding sites might still be present, but if they are, they remain inaccessible due to the unique structure of their cell wall (e.g. carbohydrate conformation, sugar density or composition). Alternatively, the antibiotics we used might not be optimal for disrupting the cell walls of these bacteria.

 To emphasize the ability of FcMBL to be used to detect the presence of a systemic pathogenic infection even when blood cultures are negative, we tested its ability to bind LPS and LTA that are major PAMP-associated toxins released by multiple species of bacteria. FcMBL was able to detect both LPS and LTA from all 6 bacterial species tested in both buffer and blood, although detection sensitivity was consistently higher in buffer. In addition, we explored whether FcMBL binds to the antigenic PAMPs, LAM, PIM _1,2_, and PIM _6_ from *M. tuberculosis* H37Rv because these are active virulence factors associated with TB pathogenesis, and hence, they are critically important targets for point-of-care diagnostic and vaccine applications ^35– 38^. We found that FcMBL can detect LAM and PIM _6_, but not PIM _1,2_, in buffer, urine, and blood; this difference in binding is likely due to the fact that PIM _1,2_ has 4 fewer branched mannose residues than PIM _6_^39^.

 Preliminary viral antigen detection in the ELLecSA was encouraging, demonstrating FcMBL binding to 8 of 10 (80%) species. We screened these viral antigens at a range of 0.1 µg/ml to 10 µg/ml that is within or below the range at which viral proteins are known to induce an immune response in vaccines ^40– 42^.

While the FcMBL ELLecSA cannot distinguish between different types of infections, we have previously shown that it can be used to rapidly (<1 h) detect the presence of blood infections in whole blood samples from patients suspected of sepsis regardless of whether or not they have positive blood cultures ^23^. Use of the FcMBL ELLecSA in conjunction with other tools, such as C-reactive protein (CRP) and Procalcitonin (PCT), could help inform and assist the physician in deciding if there is an infection, whether hospitalization is critical, or whether antibiotics should be administered when a patient first enters a care center. In addition, we have found that we can combine the FcMBL capture of PAMPs with additional molecular diagnostic tools, such as PCR, to distinguish different pathogen types based on molecular composition of the samples ^22^.

In summary, FcMBL’s ability to both bind to numerous types of infectious pathogens and capture many of the cell wall PAMPs released by these microbes when treated with antibiotics in complex biological fluids, further demonstrates the potential value of using FcMBL capture for rapid detection of bloodstream infections, even when blood cultures are negative. As a result of extending our FcMBL-based ELLecSA studies to a broader range of different pathogens, we determined a higher (87%) binding efficiency than that observed in preliminary studies. We also now better understand how FcMBL interacts with different bacterial strains of the same species when mechanically disrupted or fragmented by antibiotics. To our knowledge, this is the broadest range and largest number of pathogens and PAMPs that have been shown can be detected by a single blood opsonin or lectin. The ability of FcMBL to detect cell wall fragments also synergizes well with standard of care antibiotic therapy, and it’s broad-range pathogen capture and detection can be leveraged to develop a wide range of infectious disease diagnostics, therapeutics, and vaccines. 

## Methods

### Pathogen sources

Bacteria, fungi, viral antigens, parasites, and bacterial cell wall antigens were obtained from a multitude of sources which include: Abcam (Cambridge, USA), AERAS (Rockville, USA), American Type Culture Collection (Manassas, USA), Biodefense and Emerging Infections Resources (BEI Resources) (Manassas, USA), Boston Children’s Hospital (Boston, USA), Brigham and Women’s Hospital Crimson Biorepository (Boston, USA), Hospital Joseph-Ducuing (Toulouse, France), Sigma-Aldrich (St. Louis, USA), Sino Biological (Beijing, China), and The Native Antigen Company (Oxford, United Kingdom).

In addition, the following defined strains were used in this study: *Streptococcus pneumoniae* 3 (ATCC 6303), *Streptococcus pneumoniae* 19A (ATCC 700674), *Escherichia coli* 41949 (Multiple O antigens:H26) (Crimson Biorepository), and *Escherichia coli* RS218 (NMEC O18:H7) (Kindly provided by James R. Johnson from the University of Minnesota). LPS from *Serratia marcescens* (L6136) *, Klebsiella pneumoniae* (L4268), *Salmonella enterica* serovar enteritidis (L6011), and LTA from *Enterococcus hirae* (L4015), *Staphylococcus aureus* (L2515), and *Streptococcus pyogenes* (L3140) were purchased through Sigma-Aldrich (St. Louis, USA). *Mycobacterium tuberculosis* H37Rv components, which include lipoarabinomannan (LAM, NR-14848) and phosphatidylinositol mannoside 1,2 and 6 (PIM _1,2_, NR-14846 and PIM _6_, NR-14847), were obtained from BEI Resources (Manassas, USA). Parasites, *Trichomonas vaginalis* (TV01-1000) and *Plasmodium falciparum* Circumsporozoite protein (ab73857), were purchased from The Native Antigen Company (Oxford, UK) and Abcam (Cambridge, USA), respectively. Viral antigens: Chikungunya E1 (CHIKV-E1), Dengue serotype 1 VLP (DENV1-VLP), Ebola GP1 (EBOVKW95-GP1-100), Tick-borne Encephalitis NS1 (TBEV-NS1-100), and Zika lysate (ZIKV-LYS-100) were purchased from The Native Antigen Company (Oxford, UK). Cytomegalovirus (CMV) was kindly provided by Brigham & Women’s Hospital (Boston, USA). Influenza H1N1 HA (11055-VNAB), Influenza H1N1 NA (11058-VNAHC), and Respiratory syncytial virus (RSV) glycoprotein g (11070-V08B2) were purchased from Sino Biological (Beijing, China). Human immunodeficiency virus (HIV) gp120 (ab174070) was purchased from Abcam (Cambridge, USA).

### Preparation of bacteria

Bacteria were subcultured in RPMI (Thermo Fisher Scientific, USA) 10 mM glucose to a McFarland of 0.5 (equivalent to 10 ^8^ CFU/ml) (Becton Dickinson, USA). Bacteria were grown to this logarithmic phase to ensure cell viability, and RPMI was used because it does not contain interfering MBL-binding nutrients, such as yeast extract. The culture was then split—live bacteria were kept on ice while the other half were fragmented. Fragmented bacterial PAMPs were generated using antibiotics or mechanical disruption. Antibiotic treatment included the appropriate use of one of the following: cefepime (NDC 25021-121-20), ceftriaxone (NDC 60505-6104-4), meropenem (NDC 63323-507-20), amikacin (NDC 0703-9040-03), gentamicin (NDC 63323-010-02), or vancomycin (NDC 0409-4332-49), at ≤1 mg/ml for ≥4 hours at 37°C 225 rpm. Mechanical disruption consisted of bead mill treatment at 30 Hz for 10 min using 0.1 mm zirconia/silica beads (BioSpec Products, USA) in a Mixer Mill MM 400 machine (Verder Scientific, Inc., USA). Testing by FcMBL ELLecSA was performed on titers of live bacteria at ≤10 ^7^ CFU/ml, and on the same concentration of bacteria after fragmentation. LPS endotoxin from Gram-negative bacteria was quantified using a limulus amebocyte lysate (LAL) assay ([Endosafe®] Charles River Laboratories, USA).

### Preparation of fungi, viral antigens, parasites, and bacterial cell wall antigens

Fungi species were primarily subcultured in RPMI 10 mM glucose; however other media, such as potato dextrose broth (Teknova, USA), were used to facilitate growth. In these cases, the fungal cells were pelleted at 3,000 × *g* for 5 minutes at 22°C (Eppendorf 5424, USA), washed 3x in 50 mM Tris-HCl, 150 mM NaCl, 0.05% Tween-20, 5 mM CaCl _2_, pH 7.4 (TBST 5 mM CaCl _2_) (Boston BioProducts, USA) to remove residual growth media, and then resuspended in TBST 5 mM CaCl _2_. Testing by FcMBL ELLecSA was performed on titers of live fungi at ≤10 ^7^ CFU/ml. Purified or inactivated viral antigens, parasites, and bacterial antigens were resuspended or diluted in TBST 5 mM CaCl _2_ for testing directly by FcMBL ELLecSA. *Trichomonas vaginalis* was screened at 0.46 µg/mL and *Plasmodium falciparum* Circumsporozoite protein was screened at 50 µg/mL. Concentrations of viral antigens tested are indicated in the legend of Figure 7.

### Antibodies

Anti-protein A (catalog number ab19483) was purchased from Abcam (Cambridge, USA).

### Biological reagents

Fresh whole human blood (sodium heparin) was purchased from Research Blood Components, LLC. (Boston, USA), and normal single donor human urine (IR100007) was purchased from Innovative Research Inc. (Novi, USA).

### FcMBL ELLecSA

The key metric used to quantify direct FcMBL binding to pathogen-associated molecular patterns (PAMPs) from bacteria, fungi, viral antigens, parasites, and bacterial cell wall antigens is a 96 well ELLecSA, which has been previously published ^23^. The assay uses FcMBL coated superparamagnetic beads (1 µm MyOne Dynabead [Thermo Fisher Scientific, USA]) where FcMBL, biotinylated at the N termini of the Fc protein using an N-terminal amino-oxy reaction, is coupled to streptavidin beads in an oriented array ( Figure 1A). Each sample was screened using 100 µl or 200 µl of test sample added to 900 µl or 800 µl of assay solution respectively, which contains 5 µg of the FcMBL beads at 5 mg/ml and 10 mM glucose in TBST 5 mM CaCl _2_ to total 1 ml (50 µl heparin is added if testing blood). PAMPs in the test sample are captured by FcMBL for 20 minutes at 22°C 950 rpm in a plate shaker (Eppendorf, USA). Using an automated magnetic-handling system (KingFisher ^TM^ Flex [not shown]) (Thermo Fisher Scientific, USA), captured PAMPs are washed two times using TBST 5 mM CaCl _2_, and detected with human MBL (Sino Biological, China) linked to horseradish peroxidase (MBL-HRP). Non-specific MBL-HRP is removed by 4 washes in TBST 5 mM CaCl _2_, and PAMPs are quantified with 1-step ultra tetramethylbenzidine (TMB) substrate (Thermo Fisher Scientific, USA). Finally, the reaction is quenched with 1 M sulfuric acid and results are read at the optical density 450 nm wavelength. Quantification of bound PAMPs is determined using a standard curve generated using yeast mannan (catalog number M3640, Sigma-Aldrich, USA) in TBST 5 mM CaCl _2_—a known target for MBL (1 ng/ml mannan = 1 PAMP unit) ^43^. PAMP units are multiplied back by the dilution factor of the test sample volume to give PAMPs/ml. Previously, a receiver operating characteristic comparison was performed for a small pilot sepsis patient study in which sepsis blood was analyzed versus non-infected controls to determine an optimal ELLecSA threshold of 0.45 PAMP units ^23^. Therefore, in this study we define and report FcMBL binding to a sample as having ≥5 PAMPs/ml. To confirm specificity of FcMBL binding, a negative control (FcMBL null) was used alongside FcMBL in the ELLecSA. FcMBL null was engineered by introducing two residue mutations, E347A and N349A, into aktFcMBL (GenBank accession: KJ710775.1) to remove functional binding of the CRD of MBL. FcMBL null was purified and used to coat beads in the same fashion as FcMBL described above for direct comparison. FcMBL null beads did not support any binding to yeast mannan, and supported less than half of binding to *S. aureus* compared with FcMBL, as the Fc portion of FcMBL binds *S. aureus* protein A ^22^ (Supplementary Figure 1) ^29^.

### Statistical analysis

Data analyses on FcMBL binding to Gram-positive and Gram-negative live and fragmented bacterial isolates was performed using the statistical *R* language. Categorical variables are described as frequency (percentage). Comparisons between nominal dichotomous variables were performed with Pearson’s chi-square, when all contingency table cells were >5. Results were deemed as statistically significant when the null hypothesis could be rejected with >95% confidence. An unpaired two-tailed t-test was performed on FcMBL and FcMBL null binding to *S. aureus* using GraphPad Prism 7.0b (OS X). A p-value of < 0.05 was determined to be statistically significant. Dataset analysis is indicated in the figure legends.

### Scanning electron microscopy

For visualization of live and fragmented bacteria on FcMBL beads, bacteria were captured with 128 nm FcMBL beads (Ademtech, France), spun down onto 13=-mm coverslips and fixed with 2.5% glutaraldehyde in 0.1 M sodium cacodylate buffer (Electron Microscopy Sciences, USA) for 1 hour. Cover slips were incubated in 1% osmium tetroxide in 0.1 M sodium cacodylate (Electron Microscopy Sciences, USA) for 1 hour. Ascending grades of ethanol dehydrated the sample before being chemically dried with hexamethydisilazane (Electron Microscopy Sciences, USA). Samples were then placed in a desiccator overnight. Dried samples were mounted on aluminum stubs, sputter-coated with a thin layer of gold particles, and imaged using a Zeiss Supra55VP microscope.

## Data availability

### Underlying data

Data for this study on the broad-spectrum capture of clinical pathogens using engineered Fc-mannose-binding lectin (FcMBL) enhanced by antibiotic treatment are available from OSF. DOI: https://doi.org/10.17605/OSF.IO/GW4X7 ^29^.

### Extended data

**Supplementary figure 1. FcMBL and FcMBL null binding to *S. aureus*.** 10 ^6^ CFU/ml live *S. aureus* without and with treatment of 1 µg/ml anti-protein A, detected by ELLecSA. Anti-protein A antibody blocks the Fc-mediated binding of FcMBL null but has no significant effect on FcMBL binding. **p-value < 0.01; *p-value < 0.05; unpaired two-tailed t-test; NS: not significant. DOI: https://doi.org/10.17605/OSF.IO/GW4X7 ^29^.

Data are available under the terms of the Creative Commons Zero "No rights reserved" data waiver (CC0 1.0 Public domain dedication).
