# Supplementary material for: Broad-spectrum capture of clinical pathogens using engineered Fc-mannose-binding lectin enhanced by antibiotic treatment
Source: F1000Res. 2019 Jan 25;8:108. [Version 1] doi: 10.12688/f1000research.17447.1 (PMC6544136; doi:10.12688/f1000research.17447.1)
Supplement: Peer review history

# Peer review: Reviewer response for version 1

**DOI:** 10.5256/f1000research.19079.r47919

**Published:** 2019-05-29

**Authors:** Anna Swierzko

**Affiliations:** 1 Laboratory of Immunobiology of Infections, Institute of Medical Biology, Polish Academy of Sciences, Lodz, Poland

Dr B. T. Seiler and co-workers present the next application of magnetic beads coated with genetically engineered form of the human MBL CRD linked to IgG Fc domain (FcMBL beads). Previously, the same group of researchers reported usefulness of that invention (in a form of biospleen or extracorporeal hemoadsorption device) in the efficient clearance of microorganisms’ cells as well as their PAMPs from blood. The presented manuscript is the valuable extension of work by Cartwright *et al.* (2016) ^1^, where FcMBL-beads were used for the rapid detection of broad spectrum pathogens in the whole blood (ELLecSA). The authors demonstrated interaction of FcMBL with bacteria, fungi and parasites. The authors confirmed also their finding that antibiotic treatment or mechanical disruption increases FcMBL-binding. No case of decreased binding after antibiotic treatment or bead milling was observed, however some isolates, even after disruption were still not recognised by FcMBL. Among them there were *Enterococcus faecalis* and *Proteus* isolates. These finding are in agreement with previous reports by Geiss-Liebisch *et al.* (2012) ^2^ and Man-Kupisinska *et al.* (2018) ^3^. Geiss-Liebisch demonstrated that wall teichoic acids and rhamnopolysaccharide of *E. faecalis* prevent peptidoglycan from MBL binding. Whereas Man-Kupisinska, despite observed common interaction of MBL with the conservative inner core of numerous enterobacterial lipopolysaccharides, did not find MBL interaction with 42 of 70 tested *Proteus vulgaris*, *Proteus mirabilis* and *Proteus penneri* isolated LPSs. The authors should consider including this information into the manuscript.

 The authors also presented ability of FcMBL to capture isolated bacterial and viral antigens. They also described detection of *M.tuberculosis* mannosylated LAM in spiked blood and urine. Surprisingly, the sensitivity in urine was markedly reduced, not only in comparison to buffer but also in comparison to blood. Previously the authors described that there are inhibitors (probably immunoglobulins) in joint fluids preventing FcMBL interaction with bacteria. Treatment with proteases and hyaluronidase was proposed to improve pathogen capture. Since rapid and sensitive method of LAM detection in urine could be of big importance in TB diagnosis, it would be important to identify these inhibitors.

 This is a very important and well done study. FcMBL-coated beads are demonstrated as a sensitive alternative to the time consuming and often false negative blood culture. It can also be a fantastic tool, helpful in the screening and simple identification of MBL ligands (for example – after SDS-PAGE-WB and immunostaining with PAMP-specific antibodies). 

 Minor points

1. Mannosylated LAM *Mycobaterium tuberculosis* H37Rv strain should be described as “ManLAM”
2. Page 4: probably should be “including multiple isolates” instead of “including multiple species”
3. On the page 4, referring to Table 1 - the authors stated that they tested FcMBL binding to 12 different species of fungi. However , in Table 1 only 3 isolates belonging to 3 species are listed and in Figure 4 – results of FcMBL interaction with 13 fungi isolates is presented. It is confusing.
4. On page 3 the Authors stated that pathogens were treated with antibiotic within 4 hours, however according to the method section – “bacteria were treated with antibiotics for ≥4  hours”. It should be clarified.

I have read this submission. I believe that I have an appropriate level of expertise to confirm that it is of an acceptable scientific standard.

## References

[1] CartwrightMRottmanMShapiroNISeilerBLombardoPGaminiNTomolonisJWattersALWaterhouseALeslieDBolgenDGravelineAKangJHDidarTDimitrakakisNCartwrightDSuperMIngberDE: A Broad-Spectrum Infection Diagnostic that Detects Pathogen-Associated Molecular Patterns (PAMPs) in Whole Blood. *EBioMedicine*.2016;9: 10.1016/j.ebiom.2016.06.014 217-227 10.1016/j.ebiom.2016.06.014 27333027PMC4972566

[2] Geiss-LiebischSRooijakkersSHBeczalaASanchez-CarballoPKruszynskaKReppCSakincTVinogradovEHolstOHuebnerJTheilackerC: Secondary cell wall polymers of Enterococcus faecalis are critical for resistance to complement activation via mannose-binding lectin. *J Biol Chem*.2012;287(45) : 10.1074/jbc.M112.358283 37769-77 10.1074/jbc.M112.358283 22908219PMC3488052

[3] Man-KupisinskaASwierzkoASMaciejewskaAHocMRozalskiASiwinskaMLugowskiCCedzynskiMLukasiewiczJ: Interaction of Mannose-Binding Lectin With Lipopolysaccharide Outer Core Region and Its Biological Consequences. *Front Immunol*.2018;9: 10.3389/fimmu.2018.01498 1498 10.3389/fimmu.2018.01498 30008719PMC6033962

# Peer review: Reviewer response for version 1

**DOI:** 10.5256/f1000research.19079.r43622

**Published:** 2019-01-28

**Authors:** Damon Eisen

**Affiliations:** 1 James Cook University, Townsville, Australia; 2 Townsville Hospital and Health Service, Townsville, Australia

The authors, who are the inventors of the FcMBL capture assay, have undertaken rigorous experimental work to produce a clinically oriented paper that extends the range and numbers of microbes tested in prior publications.

 The current publication describes the detection of bacteria, fungi, parasites and viral antigens by the enzyme-linked lectin sorbent assay (ELLecSA). Microbiologically relevant quantities of these microbes were tested *in vitro *and high rates of detection by ELLecSA are reported. As before, fragmentation of bacteria using antibiotics and physical fragmentation increased the numbers of bacteria detected.

 The mechanisms of this increased sensitivity of detection were explored by the quantification of FcMBL-binding to the major virulence determinants of gram-negative and gram-positive bacteria. Greater sensitivity of binding to LPS was shown compared with LTA. This result provides some explanation for FcMBL's failure to detect *E. faecalis.*

 The current publication does not include any further investigation of patients with the sepsis syndrome relying on the author's prior work to imply that a broader range of bacterial causes of sepsis will be detected. This is a reasonable assumption. 

 Further work using the same *in vitro* techniques could incorporate assessment of FcMBL's detection of rickettsia, leptospires and treponemes. These bacteria all cause diseases that are predominantly diagnosed by serology. Expedited diagnosis of blood stream or central nervous system infection would be clinically beneficial and direct appropriate antimicrobial treatment in patients with appropriate epidemiological characteristics. This could be particularly relevant to use of FcMBL technology as part of biodefence. 

 As the authors emphasise, the use of FcMBL can only indicate the presence of a microbial pathogen in the bloodstream due to the non-specific characteristics of binding to PAMPs. A positive ELLecSA may provide clinicians confidence that a patient presenting with the sepsis-syndrome has an infectious cause. Further testing in such patients will give additional information on the validity of the test and may provide additional encouragement for its incorporation in a microbiological testing algorithm.

I have read this submission. I believe that I have an appropriate level of expertise to confirm that it is of an acceptable scientific standard.
